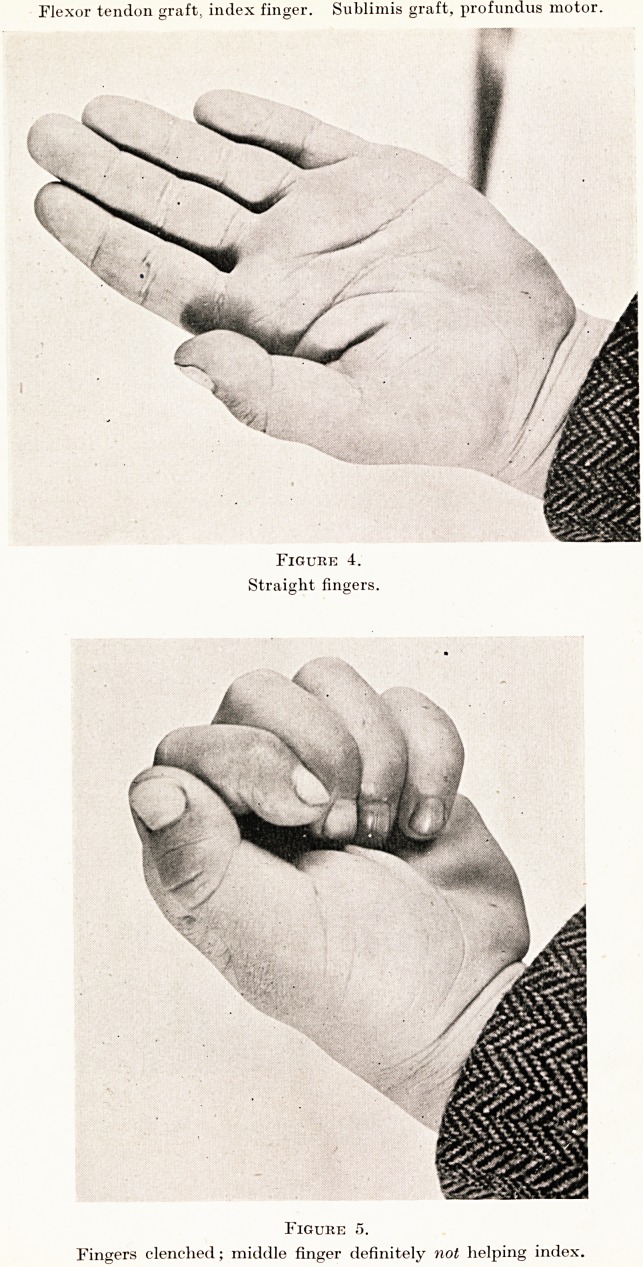# The Problem of Cut Flexor Tendons in Fingers

**Published:** 1949-07

**Authors:** A. L. Eyre-Brook

**Affiliations:** Orthopædic Surgeon, United Bristol Hospitals and Winford Orthopædic Hospital


The Bristol
Medico-Chirurgical Journal

*A Journal of the Medical Sciences for the*

West of England and South Wales

*" Scire est nescire, nisi id me
Scire alius sciret."*

JULY, 1949.

### THE PROBLEM OF CUT FLEXOR TENDONS IN FINGERS

BY

A. L. Eyre-Brook, M.S., F.R.C.S.

*Orthopædic Surgeon, United Bristol Hospitals and

Winford Orthopædic Hospital.*

Cut flexor digitorum tendons, when the cut is at the wrist, do exceedingly well if sutured with silk, although the operation is tedious and accurate reconstruction essential. When the cut is in the palm outside the flexor sheath results are fairly good. But surgeons have for long faced a real problem in treatment when the cut is in the fingers : indeed, so great are the difficulties that some surgeons have advocated amputation. It is therefore surprising to find that these cases are still sometimes dealt with by the enthusiastic casualty officer who, during his short stay in this department, rarely grasps the completeness of his failure and who often continues to view the problem as one of cobbling the cut tendons with silk of suitable strength. This practice is not only doomed to failure but usually spoils the field for subsequent surgery.

Let us consider some of the points which complicate the treatment and jeopardize the result in this common injury.

1. A partial success is a failure : because a finger which cannot act with the other fingers either gets in the way, or being held apart inco-ordinates the action of the hand. A very good result is essential. A cut flexor pollicis longus is a much simpler problem, if only for the reason that the thumb acts on its own and defective flexion is no serious disability.

2. The cut may well be infected.

3. The complicated anatomy of tendon sheaths and retaining slings has to be preserved or reproduced to get good function.

4. Suture material which is strong enough to resist the muscle pull will cause extensive foreign-body reaction and thick fibrous adhesions, unless removed as soon as it can be dispensed with.

5. The skin and subcutaneous scar of the original injury usually overlies the tendon suture and this with the suture material predisposes to a transverse bar of scar tissue. The accidental cut is always *across*, and therefore most liable to tether, the tendon ; surgical scars are usually lateral and in the length of the finger.

The surgeon who has done most towards the solution of this problem is Sterling Bunnell of San Francisco. He introduced removable stainless-steel wire sutures, which were strong enough to resist the pull of the muscle and yet caused a minimum of fibrosis during their fourteen days' stay within the tissues. He also made it clear that the best results were obtained when there was no tendon suture within the extent of the flexor tendon sheath in the finger ; this frequently makes it necessary to use a tendon graft.

*Immediate suture.* In a small series, the cut flexor profundus tendon was sutured with a stainless-steel removable wire suture and the sublimis tendon excised from the finger and palm ; this being done within a few hours of the injury. Results were not very encouraging, largely because the overlying transverse scar of the cut in the skin and subcutaneous tissue adhered to the tendon suture and formed one strong fibrous scar.

*Delayed suture*, allowing the cut to heal and the scar to mobilize before entertaining surgery on the tendon and using correctly planned skin incisions, led to somewhat better results. It has latterly only been used for cuts in the palm and the thumb, which have a much better prognosis than cuts in the finger.

*Delayed tendon grafts.* The common accident, in which the flexor tendons are cut in the finger, has of late been dealt with by delayed tendon graft. The cut is carefully cleaned, damaged skin excised and the fresh edges sutured immediately after the accident. No surgery of the tendon is undertaken until the finger has regained its full passive range of movement and the scar has become soundly healed and shows some mobility over underlying tissues. This interval is usually about six weeks. The muscle of the affected flexor profundus tendon will not atrophy as the tendon does not retract far. It either becomes attached to the sheath or is held by the lumbricales muscles : it is therefore able to act during use of the hand. A well-planned tendon graft is then carried out under the best conditions for success. The important points in the operation are as follows :

HÆMOSTASIS. Operate under ischæmic conditions provided by a baumanometer cuff after suitable pressure-exsanguination of the hand and forearm. This cuff can be kept on for well over an hour without ill effect, pressure being maintained at 200 mm. of mercury. When the suture of the tendon graft has been completed, some tidying up will be necessary and at this stage it is advisable to deflate the baumanometer cuff. Free capillary and arterial hæmorrhage occurs. The latter should be controlled by an occasional very fine ligature and the former by pressure with a swab soaked in warm saline. Even with these precautions it is still often advisable to drain the incision in the palm as any hæmatoma will ruin the result.

SKIN INCISIONS should be lateral and in the long axis of the finger : and in the creases of the palm. The oblique palm crease is the best, giving quite good longitudinal exposure.

PREPARING THE BED FOR THE TENDON. Both tendons distal to the cut are removed except for the final quarter-inch of the flexor profundus tendon, which is used to receive the distal end of the graft. The lateral finger incision cuts the two retaining pulleys on one side, near their attachment to the phalanges. These pulleys are not resutured. This allows for swelling of the tendon graft without strangulation. Repair of the pulleys will occur naturally so that their essential function is preserved, and the tendon is prevented from acting as a bow string. Any scar tissue at the site of the original cut is, of course, excised *en bloc*.

THE TENDON GRAFT. The flexor profundus tendon proximal to the cut is shortened, if necessary, to bring the suture line just proximal to the distal palm crease. The flexor sublimis tendon proximal to the cut is removed, if necessary up to the muscle belly, to provide the tendon graft of suitable cross-section. The tendon graft can be removed through a fresh incision in the forearm but usually sufficient tendon can be obtained through the palm incision. The graft should be very slightly overlength as some shrinkage does occur. (Figures 1 and 2.) The palmaris longus has been found to be a most unsuitable graft, owing to its small size. The end of the flexor profundus tendon is not " satisfied " by the cross-section offered by the proximal end of a palmaris graft and many " unsatisfied " fibres gain attachment to surrounding tissue, forming a collar of dense adhesions.

Fig. 1.

Diagrammatic representation of cut flexor tendons in finger.

Fig. 2.

Flexor sublimis graft replacing cut flexor profundus tendon; stump of flexor sublimis removed and both ends of the flexor profundus shortened.

Fig. 3.

The Sterling Bunnell method of stainless-steel wire suture. The suture can be totally removed by the " pull out " wire when the ends round the button are cut off the fourteenth day.

THE SUTURES. Suture is effected at both ends of the graft by removable stainless-steel sutures which are withdrawn on the fourteenth day. The Sterling Bunnell suture is illustrated on facing page. (Figure 3.)

Occasionally the suture line is improved by a very fine suture of 6/0 ophthalmic "mersilk". As these sutures leave a permanent foreign body, they are avoided whenever possible. If the proximal suture line can be covered with some fatty flap or the base of the lumbricalis muscle, the chances of adhesions are reduced.

CLOSURE. When the surgeon has carefully dealt with bleeding and provided a small drain, the skin is sutured. A firm pressure dressing consisting of a liberal supply of cotton-wool and two crêpe bandages completes the operation.

POST-OPERATIVE TREATMENT. The drain is removed on the second day when the hand is inspected. The wires are withdrawn proximally on the fourteenth day after cutting off the button at the distal end. To avoid any trouble when removing the wires, make a point at the time of the operation of ascertaining that they slip freely backwards and forwards and are not hampered by any kinks. This post-operative routine precludes almost all movement of the tendon during the first two weeks, the suture line being tethered to the skin. Passive movements of the fingers are encouraged once daily and active movements initiated after the removal of the stainless-steel suture. Massage of the scars, when these are thoroughly healed (three to four weeks) is useful and so also is faradic stimulation after six weeks. Occupational therapy helps a great deal in recovering active movements. In a satisfactory case a full range of movement should be obtained in about three months : results can be fairly assessed at this period as little further improvement is to be expected. On the whole it appears that children and young people give the best results.

The suture line has occasionally been exposed at a later operation for adhesions. It has then been found that the best sutures show merely a fusiform enlargement, while in those where the graft is much smaller in cross-section than the flexor profundus tendon there is a collar of thick adhesions from what may be termed "unsatisfied" fibres of this tendon. The graft always appeared white and glistening and showed no yellow degenerative changes.

It will be noted that only the flexor profundus tendon has been reconstructed by suture or grafting and that the flexor sublimis tendon has been removed *in toto*. This practice is now invariable as one finds good results sufficiently difficult to obtain when concentrating on one tendon without adding the complicating factor of having two tendon sutures both requiring the opportunity to swell and to form fusiform unions, usually at approximately the same level. Adhesions are consequently much greater where both tendons are preserved, and the results are inferior. It is quite surprising how excellent the function can be in a finger which has lost the action of the flexor sublimis. The illustrations demonstrate that the flexion is almost complete in both joints. (Plate VIII.) The patient was shown at the clinical meeting in December. This boy can demonstrate perfect function by allowing his middle finger to assist in flexing the index finger, the former lying slightly posterior to the latter. In the photograph no such trick is being used.

A not uncommon lesion is division of the flexor profundus tendon distal to the insertion of the flexor sublimis. Here the disability is very much less than when both tendons are severed. The middle joint of the finger, designed to bend well beyond the right angle, continues to function normally : while the distal joint, which only bends to considerably less than a right angle, ceases to function. Some surgeons do repair the flexor profundus tendon in such cases but there is a definite risk of impairing the perfect flexor sublimis function. There is a great deal to be said for leaving such cases entirely alone. Fixation of the distal joint at 135 deg. is a safe procedure if it is felt that surgery is called for to improve function.

The surgery for cut flexor tendons in the fingers provides a very exacting and interesting study. It certainly warrants the skill of a fully trained surgeon and excellent results can be obtained. The pessimistic conclusion that amputation of the finger is called for is quite unwarranted, as also is the optimistic conception that the treatment lies within the province of the surgery undertaken in the casualty department. The finger is one of the smallest members but the whole arm exists solely to serve the thumb and four fingers. How much of our time and effort is directed towards getting these prehensile members into action !

### PLATE VIII

Flexor tendon graft, index finger. Sublimis graft, profundus motor.

FIGURE 4.
Straight fingers.

FIGURE 5.
Fingers clenched; middle finger definitely *not* helping index.

## Figures and Tables

**Fig. 1. f1:**
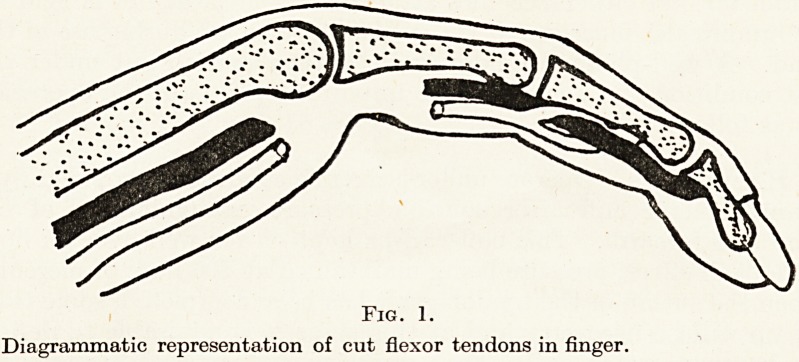


**Fig. 2. f2:**
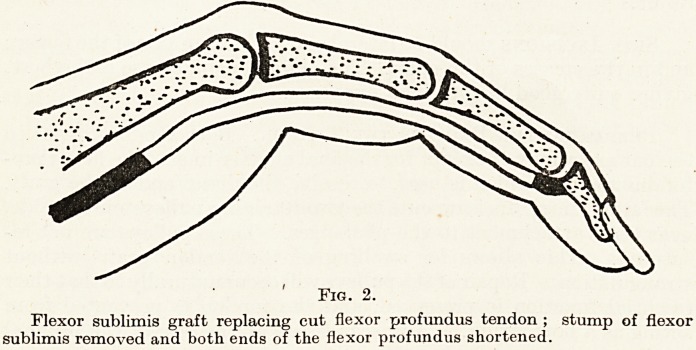


**Fig. 3. f3:**
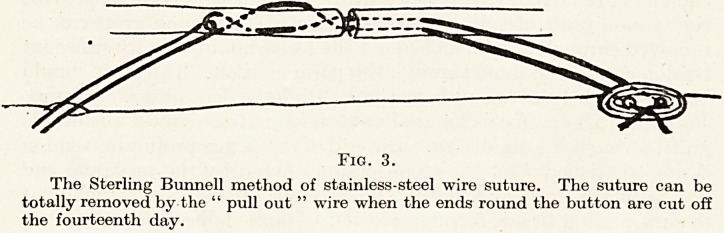


**Figure 4. Figure 5. f4:**